# The Intergenerational Transmission of Trauma, Adverse Childhood Experiences and Adverse Family Experiences: A Qualitative Exploration of Sibling Resilience

**DOI:** 10.3390/bs15020161

**Published:** 2025-02-01

**Authors:** Miriam Riaz Nichol, Lee John Curley, Pamela Jane Sime

**Affiliations:** 1 School of Psychological Sciences & Health, University of Strathclyde, Glasgow G1 1XQ, UK; 2 School of Health & Life Sciences, Glasgow Caledonian University, Glasgow G4 0BA, UK; lee.curley@gcu.ac.uk; 3 School of Health & Social Care, Edinburgh Napier University, Edinburgh EH11 4BN, UK; pamelajane.ritchie@napier.ac.uk

**Keywords:** intergenerational trauma, adverse childhood experiences, adverse family experiences, resilience, coping, case study

## Abstract

The Adverse Childhood Experiences (ACEs) and Adverse Family Experiences (AFEs) frameworks have been employed extensively in research. However, to date, no such studies have considered both frameworks concurrently, nor have they explored the similarities and differences in resilience between siblings with ACE- and AFE-exposed parent(s). Doing so could, perhaps, establish the ways in which adversity is transmitted through generations and subsequently identify what trajectories to address in potential interventions. Thus, the objectives of this study were to examine the experiences of families with intergenerational ACE and AFE trauma, and to ascertain what influences similarities and differences in sibling resilience. The thematic analysis of two family case studies (*n* = 6) proposed a narrative encompassing fear, relationships and challenging trauma. Findings demonstrated the influence of neighbourhood violence which appeared to initiate this narrative. The fear associated with neighbourhood violence and maltreatment in early childhood appeared to influence both the parent–child bond and intimate relationships. Finally, participants demonstrated the protective properties of dissociation and sibling parentification, exhibiting their relationship with resilience. Investigators suggested neighbourhood violence be considered in the ACE questionnaire to inform current and future policies, and to safeguard the needs of families affected by intergenerational trauma.

## 1. Introduction

Scholars of the 21st century have proliferated the trauma literature seeking to ascertain the role of childhood maltreatment (CM) on life course trajectories (Howell et al., 2021). Some examples of childhood adversities explored include natural disasters (Meltzer et al., 2021), terrorism and war (Samara et al., 2020) and severe pediatric illness (Stanzel & Sierau, 2022). Within a Scottish context, Adverse Childhood Experiences (ACEs), which includes CM and household stressors (HS), and Adverse Family Experiences (AFEs), which focus on an environmental perspective, have become a grave public health issue, with recent statistics demonstrating their growing prevalence (Marryat & Frank, 2019; Scottish Government, 2024). For instance, 71% of adults in Scotland have reported having experienced at least one ACE, while 15% have reported four or more (Scottish Government, 2024). Similar reports have also evidenced the disproportionately high rates of violence within deprived Scottish communities, particularly for young males (Fraser et al., 2024). This is cause for concern as studies have established that cumulative ACEs and AFEs increase the risk of psychopathology and suicide ideation (Jia et al., 2020), substance use and dependency (Hughes et al., 2020), criminality (Basto-Pereira et al., 2022) and the intergenerational transmission of trauma (ITT) (Hughes et al., 2017). As such, researchers have sought to ascertain the severe and perpetuating intergenerational threats of ACEs and AFEs (Swords et al., 2024) so as to inform policy and preventative interventions, including those that seek to foster resilience (Ortiz, 2019). Intergenerational trauma pertains to the phenomenon whereby adversity experienced in one generation, namely in childhood, can perpetuate to subsequent generations (Keaney et al., 2024). Although often used interchangeably with ‘historical trauma’ and ‘transgenerational trauma’, the former is distinguished through its relation to systemic trauma (Cerdeña et al., 2021), and the latter with epigenetics (Švorcová, 2023). Individual traumas that occur on a smaller scale (e.g., interpersonal violence) do not necessarily fall within the remit of historical trauma (Keaney et al., 2024), thus demonstrating the use of the term ‘intergenerational trauma’ within this context throughout this paper.

Although not novel in their assessment of cumulative risk (Rutter, 1980), Felitti et al. (1998) expanded upon previous efforts (e.g., Kempe et al., 1962) becoming the most prominent study examining CM. The seminal work of Felitti and colleagues (1998) acknowledged the prevalence of both CM and HS, demonstrating that they seldom occur in isolation, and are, instead, experienced concurrently (Felitti et al., 1998). The dose–response relationship they identified developed the conceptualization of CM, constituting a paradigm shift within the literature, by stressing the cumulative and chronic nature of ACEs on later health outcomes (Felitti et al., 1998; Struck et al., 2021). Consequently, a succinct index of ACEs encompassing five subtypes of CM (i.e., verbal/emotional, physical and sexual abuse; emotional and physical neglect) and the exposure to various forms of HS (i.e., parental separation/divorce, witnessing intimate partner violence (IPV); substance abuse; mental illness and incarceration) (Cerdeña et al., 2021; Dube et al., 2001a, 2001b) were proposed, and have since become employed extensively in research (Narayan et al., 2022).

Nevertheless, the ACE framework has faced controversy for omitting contextual and familial hardships (Cronholm et al., 2015), which scholars suggest compound the effects of ACEs (Camacho & Henderson, 2022). Subsequently, the AFEs measurement was developed, adopting items from the ACE questionnaire (excluding emotional/physical and sexual trauma) and supplementing them with four new items (i.e., death of a parent, low income, racial abuse and neighbourhood violence) (Kasehagen et al., 2018). Although researchers have emphasized that ACEs and AFEs do not determine specific outcomes, the graded relationship between the number of ACEs and AFEs encountered, and the frequency and severity of risk and health outcomes have been evidenced (Felitti et al., 1998; Kasehagen et al., 2018). With scholars applying a multitude of frameworks to explore this association, including that of attachment theory (Bowlby, 1980).

Attachment theory (AT) (Bowlby, 1980) posits the repeated dyadic interactions between infant and primary caregiver act as a prototype for interpersonal behavior, emotions and intimate relationships (Bosmans et al., 2020). Children who receive consistent and supportive care are likely to develop secure attachments, allowing them to seek parental comfort when distressed (Bosmans et al., 2020; Cooke et al., 2019). By contrast, children raised in dysfunctional environments characterized by adversity, are likely to perceive their primary caregiver as inconsistent, insensitive and untrustworthy, contributing to insecure (i.e., avoidant and anxious) and disorganized attachments (Bowlby, 1982; Paetzold & Rholes, 2020).

One relationship that AT can provide a theoretical framework for is the associations between maternal ACEs, problematic parenting and the ITT (Kim et al., 2021). Studies have demonstrated that ACE-exposed mothers are likely to adopt authoritarian parenting characterized by hostility and disengagement (Hanetz-Gamliel & Dollberg, 2022). Specifically, a maternal history of maltreatment is a strong predictor of the mother becoming neglectful and perpetrating physical and psychological abuse of her own children, contributing to the ITT and ACEs (Letouneau et al., 2019; Mitani, 2022). Research has also established that mothers with a history of ACEs exhibit limited sensitivity, diminished maternal self-efficacy, and are often emotionally unavailable to their children (Jiwani et al., 2022; Rowell & Neal-Barnett, 2021), all of which contribute to an insecure mother-infant attachment (Racine et al., 2018), which appears to mediate the effects of maternal trauma on child developmental trajectories (i.e., health, learning and behavior) (Cooke et al., 2019).

Scholars have long debated the reasoning for this relationship (Hanetz-Gamliel & Dollberg, 2022). For instance, some suggest ACE-exposed mothers are disposed to experiencing affect dysregulation, in which the ability to regulate/respond to negative emotional stimuli is impaired (Dvir et al., 2014). This disposition tends to exacerbate when parental strain arises (Ahlin & Atunes, 2016), including neighbourhood violence (NV). There is evidence that has suggested mothers residing in deprived, high-crime communities exhibit frustration and hopelessness derived from their inability to protect their children from violence (Ahlin & Atunes, 2016). Such parenting challenges can elicit painful memories relating to trauma, especially if supplemented with an insecure maternal attachment (Mikulincer & Shaver, 2019; O’Brien et al., 2019). As such, the mother may find detaching from and differentiating between her ACEs and parental strain difficult, compromising sensitive and responsive parenting and instead fostering the development of insecure parent–child attachments (Hanetz-Gamliel & Dollberg, 2022). Hence, communities saturated with violence and deprivation not only contribute to and perpetuate the ITT through chronic violence exposure, but also through the development of insecure attachments and problematic parenting (McCoy et al., 2015).

Over the past two decades the operationalization of resilience has developed significantly from focusing on the idea that resilience is a unique quality of invulnerability, to progressing to a modern developmental systems perspective (Masten, 2018). The latter defines resilience as the capacity and processes by which a dynamic system acclimatizes effectively to adversity through perseverance to facilitate positive development (Rava et al., 2023). One way in which resilience mitigates the negative effects of adversity is through influencing the coping strategies an individual might adopt, either exacerbating or ameliorating the stress response (Hall et al., 2021). The resilience literature acknowledges two coping styles: problem-focused coping (PF) and emotion-focused (EF) coping (Sheffler et al., 2020). PF coping pertains to the identification and active management of a stressor, fostering self-efficacy (Landy et al., 2022), whereas, EF coping refers to the elimination of negative emotional stimuli, preventing the direct addressal of a stressor (Landy et al., 2022). Following ACE and AFE exposure, an array of PF (e.g., caring for others) and EF (e.g., self-blame and dissociation) coping mechanisms are typically adopted (Solberg et al., 2023), although researchers have established that adversity is more strongly associated with maladaptive EF coping strategies (Sheffler et al., 2020). Scholars have attributed this association to the disruptions in emotion regulation, whereby, following adversity, the elimination of negative emotional stimuli is preferred (Nusslock & Miller, 2016), subsequently increasing the risk of psychiatric outcomes and substance use, both of which have been linked to low resilience (Solberg et al., 2023).

Although the literature examining the varying health and developmental outcomes of ACEs or AFEs abounds, no examinations thus far have considered ACEs and AFEs concurrently. Establishing the developmental origins of parental trauma is fundamental to determine whether specific parenting behaviors sustain intergenerational pathways of trauma that, perhaps, constitute significant risk for ACEs in subsequent generations (Narayan et al., 2022). Further, the trauma literature has demonstrated that, although siblings may share traumatic experiences, they often perceive these experiences differently (Donagh et al., 2022). Despite this well-established finding, no examinations thus far have explored what influences the similarities and differences in sibling resilience within a Scottish ACE and AFE context. This could, perhaps, be significant for identifying how adversity is transmitted to subsequent generations and to ascertain what trajectories to address in potential interventions, specific to each sibling. Thus, the present study aims to bridge the aforementioned gaps in the literature by exploring the following research questions respectively.

RQ 1: What are the experiences of families with intergenerational trauma in relation to ACEs and AFEs?
RQ 2: What influences the similarities and differences in sibling resilience across case studies?

## 2. Materials and Methods

### 2.1. Ethics and Risks

Ethical approval was acquired from Glasgow Caledonian University (GCU) School of Health and Life Sciences ethical committee [HLS/PSWAHS/23/176], with research conducted in accordance with the British Psychology Society (BPS) practice guidelines (Practice Board, 2017).

### 2.2. Design and Materials

A qualitative collective case study design (Crowe et al., 2011) was employed as, to date, researchers have taken a predominately quantitative approach (e.g., Gomis-Pomares & Villanueva, 2023; Skolnick et al., 2023). This facilitated the exploration of complex phenomena through a variety of lenses (Baxter & Jack, 2008) exposing multiple relationships (Yin, 2017). Case studies comprised one parent and two siblings, allowing the ITT to be demonstrated and sibling resilience to be explored. Investigators considered triadic interviews to observe relationships between interviewees (Polak & Green, 2016). However, participants may also be reluctant to address trauma in the presence of others (Taylor et al., 2021). Thus, individual semi-structured interviews were selected. Two interview schedules were devised with seven open-ended questions (e.g., Can you describe a memory from childhood in which you felt especially loved, safe and/or understood; Was there someone in your childhood who made a particularly positive impact; What advice would you give your younger self) and relevant follow ups (e.g., Could you tell me more?) (Supplementary Material S1). Research demonstrates the anchoring effects pertaining to closed-ended questions (Frew et al., 2003), thus, open-ended questions were selected to aid with creating a more nuanced story (Rouder et al., 2021).

### 2.3. Recruitment and Participants

To ensure the selection of data-rich case studies pertinent to the phenomena of interest, purposive sampling was employed (Campbell et al., 2020). To facilitate recruitment, posters were produced and distributed around the GCU campus and social media. The relevant literature has demonstrated four or more ACEs in a parent present particularly significant ACE risks for subsequent generations (Hughes et al., 2017). Thus, parent participants had to have four or more ACEs to be eligible for participation. Further, all participants had to be over 18 years of age and be English speaking. Participants were excluded if they came from single-child families. A recent systematic review has suggested that, amongst a homogenous population, data saturation can be met with as few as five participants (Hennink & Kaiser, 2022). Having said that, when excluding outliers, this review demonstrated most datasets reached saturation with a sample of 9–17 participants (Hennink & Kaiser, 2022). Thus, considering the findings of this review and notable time constraints, investigators sought a sample of (*n* = 9). However, despite a four month recruitment period and various efforts (i.e., snowball sampling, purposive sampling), researchers were only able to recruit two family case studies (*n* = 6), although four families (*n* = 12) initially expressed interest. The research exhibits the challenges associated with recruiting participants for studies of a sensitive nature (Adikaram et al., 2022), attributing this to the stigma and shame often supplementing trauma (Vogel et al., 2020). Nevertheless, investigators determined data saturation with 6 participants (2 case studies), as no new codes were identified, allowing appropriate comparisons to be drawn (Naeem et al., 2024).

**Figure 1:**
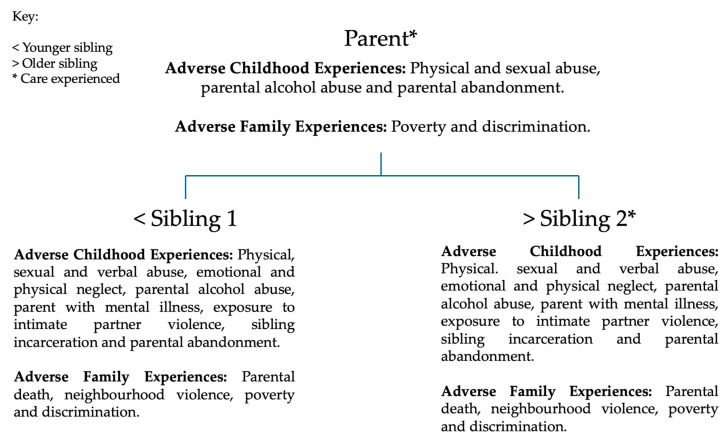
This figure demonstrates the Adverse Childhood Experiences and Adverse Family Experiences of case study one.

**Figure 2:**
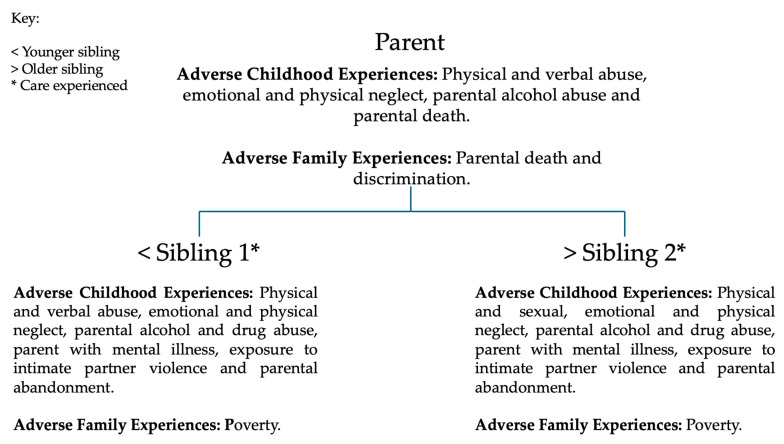
This figure demonstrates the Adverse Childhood Experiences and Adverse Family Experiences of case study two.

Participant demographics were collected; the mean age of participants was 52.66 years (SD = 14.36 years); the mean age of siblings was 46.25 years (SD = 10.90 years); and the mean age of parents was 65.50 years (SD = 13.44 years). The average number of ACEs experienced was 7.67 (SD = 2.34) and the average number of AFE experiences was 2.17 (SD = 1.17). All participant demographics are displayed in Table 1, and details of ACEs and AFEs are displayed in Figure 1 and Figure 2.

### 2.4. Procedure

Participants were provided with an information sheet, consent form and demographic sheet (for inclusion and analysis purposes) prior to their face-to-face (on GCU campus) or online interview (via Microsoft Teams). Investigators ensured participants were reminded of their rights prior to participation (i.e., data protection, confidentiality and data withdrawal). The order of interviews was based on convenience and were conducted by the principal investigator. Interviews ranged from 34 min to one hour long (M = 52.50, SD *=* 22.06) and were conducted via Microsoft Teams. In some instances, participants included details on specific trauma experiences not directly incorporated into the interview schedule (e.g., reflections on experiences of IPV). In this occurrence, investigators probed appropriately whilst monitoring body language. For instance, if disengagement or repeated self-soothing behaviors were detected (e.g., fidgeting) (Dreisoerner et al., 2021), investigators suggested pausing or terminating the session. Once completed, the recording was saved, and the participant debriefed.

### 2.5. Data Analysis

Following data collection, the principal investigator transcribed interviews verbatim into a Word document and Braun and Clarkes’ (Braun & Clarke, 2012, 2023) six stages of Thematic Analysis (TA) were utilized. Firstly, the principal investigator reviewed transcripts repeatedly to ensure data familiarization. Data pertinent to the research questions and extant literature were noted by the principal investigator and coded manually using Microsoft Word (2021, Microsoft Office), transitioning unorganized data to structured concepts (Attride-Stirling, 2001; Stocks et al., 2002).

**Figure 3:**
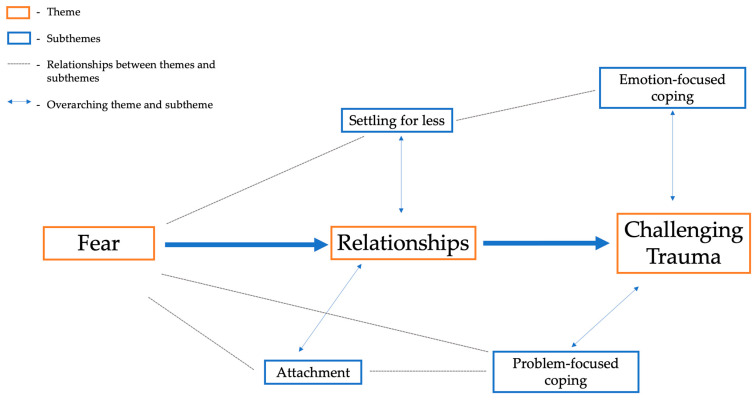
This figure demonstrates the themes and subthemes proposed following the thematic analysis of two family case studies.

Following this, coded data extracts were collated by the principal investigator to produce themes, codes were re-examined to ensure their consistency with themes and subthemes, and further amendments were made. Although the principal investigator conducted the analysis independently, the research team held regular meetings throughout the analysis process to ensure consistency and agreement. Any concerns or disagreements were discussed to consensus. Once complete, the research team began the write-up, ensuring a coherent and concise narrative of the data (Braun & Clarke, 2012, 2023). The research team were vigilant of their own preconceptions to prevent bias (Starks & Trinidad, 2007), as such an inductive approach (analysis is directed by specific objectives as opposed to theory) was adopted (Braun & Clarke, 2012). To illustrate the relationships between the proposed themes (Nowell et al., 2017), the principal investigator composed a thematic map (see Figure 3). Using Microsoft PowerPoint (2021, Microsoft Office), the principal investigator displayed each theme and their respective subtheme(s) in a way that demonstrated a coherent narrative of the dataset (Nowell et al., 2017). Dotted lines were added to exhibit the relationships between themes and subthemes clearly. Similar to the data analysis process, the research team held meetings to ensure consistency and agreement. Disagreements were resolved through consensus discussions.

## 3. Results

Using TA, three themes (i.e., fear, relationships and challenging trauma) and four subthemes (i.e., attachment, settling for less, emotion-focused coping and problem-focused coping) were proposed (see Figure 3). All themes and subthemes were observed in both case studies. Quotes were selected to best represent the ITT, with each case study exhibiting a similar, if not identical, narrative.

### 3.1. Theme 1. Fear

Theme 1 encapsulated the fear associated with NV and CM. One participant reflected on the psychological implications of losing three sons to violence.

“I suffered the loss of a further two sons who were both murdered and although I carried on living, I wasn’t really alive… I feared for my other children.”

—Parent 1, Case Study 1, Female, 76

This can be observed in subsequent generations whereby the above participants daughter attributed her brother’s murder to violations of known street codes. She reflects on the sleep disturbances that followed and highlighted vicarious trauma.

“They wanted to make sure he knew his place and that he wasn’t going to do anything unless they agreed to it… My brother was stabbed in the stomach, and he was slashed from his ear right inside his mouth with a razor blade… That caused a lot of nightmares, I was frightened to go to sleep.”

—Sibling 1, Case Study 1, Female, 53

The older sister of the above participant provides an additional perspective on the intergenerational impact of NV. She described the CM that followed, attributing this to her mother’s grief. She highlighted submissive behaviors as a defense mechanism.

“She (mother) would take the dog’s lead to me; she became aggressive after that (brother’s murder)… I don’t think any child should be petrified of their parents, but I was absolutely terrified. I would have done anything to keep the peace.”

—Sibling 2, Case Study 1, Female, 58

### 3.2. Theme 2. Relationships

Theme 2 encapsulated the relationship challenges that followed CM and violence exposure, including disturbances to the parent–child bond, represented by the subtheme (1) ‘attachment issues’, and the influence on intimate relationships, represented by the subtheme of (2) ‘settling for less’.

Attachment. Mother and daughter reflect on their experiences of parental abandonment and child sexual abuse (CSA). They suggested gender-specific attachment issues that depended upon the perpetrator’s gender. This appeared to perpetuate across the lifespan and intergenerationally.

“Not only was I suffering from abandonment, but it started a life-long hatred and mistrust in me of women… I felt much closer to my sons, but I had bonding issues with my daughters. I always felt really disappointed when I was having a daughter.”

—Parent 1, Case Study 1, Female, 75

This can be observed in subsequent generations, where the participant’s daughter expressed bonding issues with her sons, attributing this to the CSA she was subjected to.

“I remember thinking, if this is another wee boy, I’m not keeping it. I couldn’t cope with boys; I couldn’t love them; I couldn’t bond with them. I believe that’s down to the sexual abuse I experienced from my dad and brother.”

—Sibling 2, Case study 1, Female, 58

Settling for less. Participants suggested low self-esteem was cultivated through adversity, and an absence of love in childhood led to the acceptance of any affection. Both parents and two children reinforced a desensitization to violence contributed to the acceptance of IPV in adulthood.

“I didn’t deserve anything, so I settled for anything that came along. I didn’t feel worthy of a proper life. If it weren’t for my childhood and repeated abandonment, I wouldn’t have settled for the man I married… I felt it was quite normal to be beat. Although I was frightened… I felt it was a normal way of life.”

—Parent 1, Case study 1, Female, 75

This can be observed in subsequent generations in which the above participant’s daughter highlighted imitation, which appeared to subsequently facilitate further intergenerational trauma.

“I think the trauma you suffer as a child molds the person you become, and it causes you, perhaps, to choose relationships that are not quite good for you… exactly the same as what your mother and father had, therefore experiencing exactly what your mother did.”

—Sibling 1, Case study 1, Female 53

### 3.3. Theme 3. Challenging Trauma

Theme 3 illustrated two forms of coping that contributed to the similarities and differences in sibling resilience, represented by the subthemes of (1) emotion-focused coping and (2) problem-focused coping.

Emotion-focused coping. Participants reflected on coping mechanisms, whereby they stressed the efficacy of dissociation for resilient functioning.

“I found myself dissociating to the sexual abuse. When my husband was violent, I would dissociate and feel a numbness. That helped me keep going.”

—Parent 1, Case study 1, Female, 75

This can be observed intergenerationally, where the above participant’s daughter highlighted the protective properties of depersonalization (a form of dissociation) and voice-hearing experiences.

“I always managed to detach myself when traumatic things were happening, it was as if I was watching it happen as to experiencing it… The voice said to me to pretend to be a wee child. Which was ironic, because that’s exactly what I was. I was an infant. I was a baby.”

—Sibling 1, Case study 1, Female, 53

Female participants reported being introduced to substances by abusive partners, allowing the partner to intentionally increase their drug dependence through emotional manipulation, which facilitated further coercive control.

“My partner was a drug dealer, and he was violent. One day he asked if I wanted a joint and I thought fuck it… He then asked if I wanted speed… He said I needed it to take care of my kids. I eventually turned to heroin not long after and my children were placed into care.”

—Parent 2, Case study 2, Female, 56

The intergenerational effects of maternal substance use can be observed, where the participant’s son described the CSA he was subjected to in care. He expressed imitating his mother to cope with associated feelings of shame.

“He (case worker) would watch you and touch you as you showered. I couldn’t handle the shame and embarrassment… I became aggressive and addicted to coke. I turned out just like my mum.”

—Sibling 2, Case study, Male, 38

Problem-focused coping. Female participants stressed the importance of ‘sibling parentification’, where they described adopting the caregiver role to protect younger siblings from maltreatment. The protective properties of parentification were highlighted (i.e., fostering resilience and providing purpose).

“I used to get him up, change him, feed him and I missed a lot of school because of that. But I was too scared to leave him with her (mother). I knew only I could protect him… It gave me a purpose again.”

—Sibling 1, Case study 2, Female, 36

Another participant highlighted the protective properties of parentification across the lifespan, whereby they suggested parentification extends to protecting all children in the household.

“When my mother’s depression got really bad, she couldn’t look after me and my younger brother, let alone my nephew. I took over being mummy in the house. I fed him, I changed him, I wheeled him in the pram, and I cuddled him… As a child I resented it, but it made me strong and resilient. It made me a good mother.”

—Sibling 1, Case study 1, Female, 53

## 4. Discussion

Although the literature examining the health, developmental and behavioral outcomes of ACEs abound (Arnold et al., 2023), no studies thus far have considered the intergenerational effects of both ACEs and AFEs. Further, while scholars have begun to investigate the sibling relationship following ACE exposure (Donagh et al., 2022), no examinations have explored what influences the similarities and differences in sibling resilience. Thus, through the present study, the researchers sought to explore the intergenerational transmission of trauma in relation to ACEs and AFEs, while exploring sibling resilience. Following the thematic analysis of two Scottish family case studies, three themes (fear, relationships, challenging trauma) and four subthemes (attachment, settling for less, emotion-focused coping, problem-focused coping) were proposed.

Within ‘Fear’, the participants established the underlying influence of neighborhood violence (NV). To be specific, losing a child to violence was shown to compromise parenting abilities, whereby the combination of grief and trauma led to the perpetration of maltreatment of surviving children. The empirical literature has suggested the death of a child that is sudden and violent in nature can render healing difficult (Lichtenthal et al., 2013), with mothers being particularly vulnerable to experiencing severe and complicated grief (Fernández-Alcántara & Zech, 2017; Morris et al., 2019). Maladaptive cognitions of the self or others play a pivotal part in the development and perpetuation of complicated grief (Shear, 2015), impeding adaptive coping and, instead, cultivating intense emotions including anger and guilt (Komischke-Konnerup et al., 2023). Such complex patterns of grief can interfere with a parent’s ability to communicate with and support their children (Sharp et al., 2020), wherein surviving children have reported feeling overlooked (Leichtentritt et al., 2024). In the context of the present study, the empirical literature suggests ACE-exposed mothers are at risk of experiencing affect dysregulation, particularly following parental strain (e.g., loss of a child, neighbourhood violence) (Ahlin & Atunes, 2016), which can lead to insensitive, unresponsive and aggressive parenting behaviors (Racine et al., 2018). This suggests that families affected by ACEs and AFEs are at particular risk following a sudden and violent bereavement considering the maltreatment that is likely to follow, demonstrating the role of NV to the intergenerational transmission of trauma (ITT).

The emerging influence of NV was further observed in ‘Relationships’, in which participants suggested the exposure to violence in childhood contributed to a desensitization and acceptance of IPV in adulthood. This is consistent with the previous literature demonstrating that community violence exposure increases a child’s risk of violence perpetration and/or victimization later in life (Sokar et al., 2023; Butler et al., 2020). The mechanisms by which this occurs can be explained through the concept of habituation (Thompson & Spencer, 1966). Theoretically, desensitization is consistent with the well-established phenomenon of habituation, wherein the repeated exposure to a stimulus (e.g., violence) eventually leads to a diminished response (Groves & Thompson, 1970; Rankin et al., 2009). For instance, while the initial exposure to NV would elicit a naturally negative emotional response, the desensitization hypothesis argues that repeated exposure would eventually suppress such innate reactions, including emotional distress, physiological arousal and cognitive disapproval (Fanti & Avraamides, 2011; Mrug et al., 2017). The empirical literature has shown support for this hypothesis, demonstrating that chronic NV exposure can lead to an emotional desensitization through a decrease in depressive symptoms (Gaylord-Harden et al., 2017).

The pathological adaptation to violence model (Ng-Mak et al., 2004) provides insight into the acceptance of IPV. This model posits that chronic violence exposure can lead to the normalization of violence through the cognitive process of moral disengagement, which facilitates further active engagement in violent behaviors (Bacchini & Esposito, 2019). Although fewer studies have addressed the role moral disengagement plays in victimization, the available literature has suggested victims rationalize abuse through moral disengagement mechanisms (Cuaddrado-Gordillo et al., 2020), similar to the way children reconstruct experiences of maltreatment (Wang et al., 2017). The evidence suggests that the participants in the present study accepted IPV through moral disengagement mechanisms, wherein immoral behaviors are considered justifiable. Such experiences may have been transmitted from one generation to another through Social Learning Theory principles including observation and modelling (Bandura, 1973).

Within ‘Relationships’ we saw the introduction of gender-specific attachment issues, that is to say that children who experience CM can go on to develop attachment issues with either sons or daughters, depending on their perpetrator’s gender. Specifically, participants in the present study reported CSA perpetrated by the father led to attachment issues with sons and physical abuse perpetrated by the mother led to attachment issues with daughters. Although AT (Bowlby, 1980) has long-established the implications of attachment on parenting abilities, this study appeared to be the first (that the authors are aware of) to establish this phenomenon within an ACE and AFE context. A central tenet of AT and its intergenerational effects is the internalized working model (IWM) concept, whereby cognitive schemas of the self and others develop, predicated on experiences between child and caregiver (Bowlby, 1980). These cognitive schemas are believed to function outside of the conscious awareness and act as a prototype for anticipating and interpreting the intentions of others (Bowlby, 1980). Therefore, maltreated children are likely to develop negative mental representations of others which appear to affect later relationships including those with one’s own children (Bretherton & Munholland, 2016). For instance, the negative cognitive schemas developed in childhood can contribute to the development of attachment insecurity. In adulthood, this can lead to the adoption of dismissive and hostile parenting behaviors, including the avoidance of physical and emotional contact with offspring (Sette et al., 2015). Taken together, the evidence suggests participants developed negative IWMs for either men or women as representations of their perpetrators (i.e., mother or father), which led to bonding issues with specific children (i.e., sons or daughters) based on these representations. However, considering the limitations (e.g., small, gendered sample) of the present study, further examinations are necessary to explore this phenomenon with a larger, diverse sample.

Finally, ‘Challenging Trauma’ highlighted the adaptive nature of dissociation and its impact on resilience. Betrayal Trauma Theory (BTT) (Freyd, 1996) provides a theoretical framework for understanding post-traumatic outcomes. For instance, similar to the central tenets of AT (Bowlby, 1980), BTT (Freyd, 1996) states that the maltreatment perpetrated by someone who the victim depends on (e.g., parent) would likely evoke distinct outcomes. More specifically, the dependence in the victim–perpetrator relationship persuades the victim to adapt to the maltreatment in a way that maintains the parent–child attachment, thus promoting awareness inhibiting processes including freeze responses such as dissociation (Gagnon et al., 2017; Platt et al., 2016). Although the adaptive nature and protective properties of dissociation have been evidenced in the literature (Loewenstein, 2018), scholars have stressed that its adaptive function appears to be short-lived (Kumpula et al., 2011). For instance, its persistent use has been shown to increase the likelihood of revictimization by interfering with one’s ability to accurately identify safety risks (Zamir & Lavee, 2015). Therefore, these findings should be interpreted with caution, particularly considering the limitations of this study (e.g., small, gendered sample).

Finally, the phenomenon of parentification was highlighted, whereby female participants adopted the caregiver role to protect younger siblings from emotionally unavailable and violent parent(s). Minuchin’s Family System Theory (Minuchin, 1974) emphasizes the importance of hierarchical boundaries to facilitate interdependence, autonomy and positive development in children. Thus, the dissolution of hierarchical boundaries, for instance, through parentification, would in theory have adverse effects. This is supported by the previous literature, wherein the links between parentification and psychopathology have been evidenced (Khafi et al., 2014; Van Loon et al., 2017). However, other studies have suggested that parentification can foster resilience, adaptive coping and self-esteem (Borchet et al., 2015; Eskisu, 2021). Therefore, considering the mixed findings, it is possible that the sibling relationship itself may be a significant factor in mediating or moderating the aforementioned outcomes. This provides a possible insight into the present study’s findings, suggesting the positive effects of parentification can be attributed to the positive nature of the sibling relationships. However, further examinations are necessary to explore the mediating and moderating role of sibling relationship quality on the relationship between parentification and negative outcomes.

The previous literature has chosen to investigate the ITT with reference to specific ACEs or AFEs such as, violence exposure (Hashemi et al., 2022), CSA (Hornor, 2024) and psychopathology (Zhou et al., 2023). Thus, a fundamental strength of this study lies within the researcher’s decision to explore both ACEs and AFEs, and their contribution to the ITT. This decision allowed the relationships between NV (AFE) and IPV (ACE) to be demonstrated, wherein the exposure to NV in childhood desensitized individuals to IPV in adulthood. Further, the present study explored sibling resilience, highlighting the factors (e.g., emotion and problem focused coping) that perhaps contribute to the similarities and differences in sibling resilience, which to the researcher’s knowledge has not been done before within this context. It is important that research continues to explore this issue considering that siblings are likely to perceive their traumas differently, despite sharing the same experiences (Donagh et al., 2022). The promising Positive Childhood Experiences [PCEs] literature (Bhargav & Swords, 2023; Samji et al., 2024) could play a pivotal role in developing the research on the similarities and differences in sibling resilience following adversity, for instance, by ascertaining whether PCEs differ among siblings, along with other factors similar to the present study (e.g., emotion and problem focused coping), could perhaps aid with the development of tailored interventions that target specific trajectories to each sibling.

However, limitations were also present. Despite a four month recruitment period and various efforts (i.e., snowball sampling, purposive sampling), investigators were only able to recruit two family case studies, both of which had a limited number of individuals (*n* = 3) and were not culturally diverse. Prior to recruitment, investigators agreed that a sample of (*n* = 9) would perhaps be sufficient, though initially, four family case studies (*n* = 12) expressed interest. Prior to the data collection period, two case studies dropped out following informed consent. Nevertheless, investigators progressed to data analysis because of time constraints. In addition to this, investigators were unable to ascertain the impact of ACEs and AFEs from the father–son perspective as both parent participants were female. Scholars suggest females exhibit more complex patterns of CM than their male counterparts (Haahr-Pedersen et al., 2020). Thus, with limited male participants, the findings of this study cannot be applied to understanding brother–brother resilience. The difficulties surrounding male recruitment may be attributed to the gendered experience of trauma, particularly concerning relationships and experiences of IPV (Søegaard et al., 2021). For instance, recent statistics exhibit 81% of IPV cases in Scotland had a female victim and a male perpetrator (Scottish Government, 2024). By contrast, hegemonic masculine expectations encompassing invulnerability may have hampered male recruitment through associated feelings of shame (Hogan et al., 2024). Finally, recall bias must be addressed. Memories naturally deteriorate over time (Park & Festini, 2016), however, the exposure to adversity can exacerbate this process leading to autobiographical memory overgenerality (Moore & Zoellner, 2007), wherein autobiographical details are recalled inaccurately (e.g., timeline). In the context of this study, participants ranged from 36 to 75 years of age, therefore, the omission, underemphasis or exaggeration of detail is possible. Thus, to fully understand this phenomenon and to generalize findings to an overall population, further examinations are necessary. To do so, researchers must replicate this study with a greater and more diverse sample that incorporates an equal sample of males and females. It would also be beneficial to replicate this study with a younger sample to minimize memory recall bias.

## 5. Conclusions

To conclude, this study has contributed to the ACE and AFE literature, demonstrating the ITT and the potential influences that contribute to the similarities and differences in sibling resilience. This study established the underlying influences of neighbourhood violence, which appeared to initiate this narrative. Despite the literature recognizing the role of NV in the ITT, for instance, through sleep disturbances (Mrug et al., 2020), IPV perpetration (Sokar et al., 2023), re-victimization (Butler et al., 2020) and toxic stress (Lee et al., 2020), the ACE questionnaire neglects this experience. The inclusion of NV in ACE screening tools would support practitioners by increasing their ability to identify cumulative toxic stress. This is important considering toxic stress contributes to disruptions to the architecture of the developing brain which increases the risk of physical illness and psychopathology (Lee et al., 2020). Further, this addition to the ACE questionnaire would better inform policies such as the ACE Aware Initiative that helps to identify, treat and prevent trauma-induced toxic stress. To recapitulate, the present study proposed that NV initiated the ITT narrative, wherein losing children to NV triggered authoritarian parenting whereby the increased aggression from the combination of trauma and grief led to the maltreatment of surviving children. Additionally, NV exposure contributed to a desensitization and acceptance of IPV, which appeared to have intergenerational effects. Thus, to safeguard the needs of families affected by NV and other ACEs, it is important that NV is incorporated into the ACE questionnaire and other screening tools to allow policies, such as the ACE aware initiative to identify, treat and prevent trauma-induced toxic stress effectively, as supported by other scholars (Lopez et al., 2022). Finally, investigators suggest that future research ascertain whether the intergenerational transmission of trauma differs from the mother–daughter and father–son perspective, while exploring opposite sex and same sex sibling resilience.

## Figures and Tables

**Table 1:** This table demonstrates participant demographics.

Participant ID	Age	Gender	Number of ACEs	Number of AFEs
Parent 1, Case study 1	75	Female	4	2
Sibling 1, Case study 1	53	Female	10	4
Sibling 2, Case study 1	58	Female	10	3
Parent 1, Case study 2	56	Female	6	2
Sibling 1, Case study 2	36	Female	8	1
Sibling 2, Case study 2	38	Male	8	1

## Data Availability

The data presented in this study are available on request from the corresponding author due to privacy and ethical reasons.

## Supplementary Materials

The following supporting information can be downloaded at: https://www.mdpi.com/article/10.3390/bs15020161/s1.
