# Supplementary material for: The Intergenerational Transmission of Trauma, Adverse Childhood Experiences and Adverse Family Experiences: A Qualitative Exploration of Sibling Resilience
Source: Behav Sci (Basel). 2025 Feb 1;15(2):161. doi: 10.3390/bs15020161 (PMC11851674; doi:10.3390/bs15020161)
Supplement: Supplementary file 1 [file behavsci-15-00161-s001.zip › behavsci-3370218-supplementary.pdf]

| Interview Guide (Parent) | |
|---|---|
| ***Topics*** | ***Questions*** |
| Childhood and Upbringing | 1) Starting from the earliest you can remember; can you tell me about your childhood and the relationships you had with your family?<br><br>2) Was there someone in your childhood that made a positive impact? If so, please explain.<br><br>3) Was there someone in your childhood that made a negative impact? If so, please explain. |
| Parenting | 4) Can you describe a memory from childhood in which you felt especially loved, understood, or safe?<br><br>5) (**Prompt**) How has the *above* experience influenced your role as a parent/relationship with your child(ren)? |
| Protective Factors/Resilience | 6) What did you wish you had access to in childhood to help with the adversities you were experiencing? |
| Reflections | 7) If you could change one thing about your childhood, what would you change?<br><br>8) What advice would you give your younger self?<br><br>9) Is there anything else you would like to add, regardless of if you believe it is relevant? |

| Interview Guide (Siblings) | |
|---|---|
| ***Topic*** | ***Question*** |
| Childhood and Upbringing | 1) Starting from the earliest you can remember; can you tell me about your childhood and the relationships you had with your family?<br><br>2) Can you describe a memory from childhood in which you felt especially loved, understood, or safe?<br><br>3) Was there someone in your childhood that made a positive impact?<br><br>4) Was there someone in your childhood that made a negative impact? |

| | |
|---|---|
| Resilience | 5) What did you wish you had access to in childhood to help with the adversities you were experiencing? |
| Reflections | 6) If you could change one thing about your childhood, what would you change?<br><br>7) What advice would you give your younger self?<br><br>8) Is there anything else you would like to add, regardless of if you believe it is relevant? |
